# Beyond annual metrics: Linking seasonal population dynamics to vertical oyster reef growth

**DOI:** 10.1002/ece3.70238

**Published:** 2024-09-17

**Authors:** Kai Pfennings, Tom K. Hoffmann, Jan Hitzegrad, Maike Paul, Nils Goseberg, Achim Wehrmann

**Affiliations:** ^1^ Marine Research Department Senckenberg am Meer Wilhelmshaven Germany; ^2^ Ludwig Franzius Institute of Hydraulic, Estuarine and Coastal Engineering Leibniz University Hannover Hannover Germany; ^3^ Leichtweiß‐Institute for Hydraulic Engineering and Water Resources Technische Universität Braunschweig Braunschweig Germany; ^4^ Coastal Research Center Joint Research Facility of Leibniz University Hannover and Technische Universität Braunschweig Braunschweig Germany

**Keywords:** biosedimentary structure, ecosystem engineering species, *Magallana gigas*, sea level rise, terrestrial laser scanning, Wadden Sea

## Abstract

Oysters are ecosystem engineering species building reef‐like biogenic structures in temperate shallow water environments, serving as biodiversity hotspots. Recently, also their ecosystem services such as fish nursery, pollutants sink and self‐sustaining coastal protection mechanisms came into a research focus. In light of accelerated sea level rise and increasing environmental dynamics, a determination of vertical growth rates of these biosedimentary structures is paramount in assessing their resilience. This study embarked on a comprehensive survey of seasonal vertical reef growth rates using terrestrial laser scanning and related population dynamics of two intertidal reefs built by the non‐native oyster *Magallana gigas* in the Wadden Sea. We quantified median reef growth at 19.8 mm yr^−1^ for the Kaiserbalje reef and 17.5 mm yr^−1^ for the Nordland reef. Additionally, we tested the hypothesis that the seasonal variations in reef growth rates correspond to the local population dynamics, mainly the parameters of shell length and abundance which mirror delayed effects from previous spawning events. Shell growth rates were 0.03–0.06 mm d^−1^ in winter and 0.10–0.16 mm d^−1^ in summer, mean oyster abundance from autumn 2019 to spring 2022 was 627 ± 43 ind. m^−2^ and 338 ± 87 ind. m^−2^ at the Kaiserbalje and Nordland reefs respectively. Minor reef growth in the topmost reef area reflects an emerging equilibrium of the vertical reef position to actual sea level. Our findings are in accordance with growth of natural *Crassostrea virginica* reefs on the US East Coast, indicating potential resilience to actual and predicted sea level rise scenarios. Moreover, understanding local hydro‐morphodynamic feedback linked to sea level rise will be vital in predicting the three‐dimensional stability of these biosedimentary structures and habitats.

## Graphical abstract

Intertidal reefs in the Wadden Sea, built by the non‐native ecosystem engineering species Magallana gigas, have become fundamental components of the ecosystem. This study links vertical reef growth measured by terrestrial laser scanning to population dynamics on a seasonal scale, revealing that vertical reef growth rates surpass local sea level rise, similar to oyster reefs on the US East Coast and southern North Sea. It also highlights the delayed impact of recruitment events on vertical reef growth. These insights underscore the need to understand feedback mechanisms related to sea level rise and climate change to predict the resilience and potential ecological services of these biosedimentary structures.

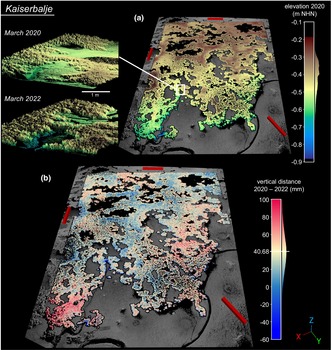

## 1 INTRODUCTION

Reef‐building oysters have emerged as prominent ecosystem engineering species in marine ecosystems since the Cretaceous period. They are instrumental in shaping temperate coastal shallow water habitats and substantially contribute to local carbonate production (Kirby, 2001; Raising et al., 2014; Shaaban et al., 1995). Recent intertidal oyster reefs worldwide are recognized for their multifaceted ecosystem services (Grabowski et al., 2012) by forming rigid three‐dimensional (3D) biogenic structure and habitat (Craeymeersch & Jansen, 2019). The reef‐building character of oysters, attributed to the cementation of successive generations (Burkett et al., 2010), is vital for the potential of oyster reefs to function as self‐sustaining green infrastructure (Dunlop et al., 2023; Morris et al., 2019; Ridge et al., 2015) that stabilize coastlines and represent biodiversity hotspots (Searles et al., 2022), especially in light of rising sea level (Ridge et al., 2015; Rodriguez et al., 2014) and accelerated climate change (Howie & Bishop, 2021). Drawing from palaeobiological studies, intertidal reefs of the oyster genus *Crassostrea* possess the ability to accrete up to several metres in response to Holocene transgressive sea level changes (Li et al., 2021). Vertical growth of recently constructed intertidal oyster reefs has been quantified up to 70–110 mm yr^−1^ in reefs of the eastern oyster *Crassostrea virginica* (Gmelin, 1791) on the US East Coast (Ridge et al., 2017; Rodriguez et al., 2014), indicating the potential to outpace projected sea level rise (IPCC, 2023). The delineation of an optimal growth zone in relation to tidal exposure time has been pivotal for assessing the vertical resilience of intertidal oyster reefs (Ridge et al., 2015) and was facilitated by advancements in spatial 3D mapping, particularly the utilization of terrestrial laser scanning (Bost et al., 2021; Fodrie et al., 2017; Ridge et al., 2015, 2017). Besides vertical growth of the oyster framework, sustainable reef accretion is a delicate balance between accumulation space in the tidal regime, biogenic shell production and sediment accumulation (Baillie & Grabowski, 2019; Coen & Humphries, 2017; Twichell et al., 2010). However, initial estimations of an optimal growth zone, characterized by 20%–40% exposure time for *C. virginica* with both lower (10%) and upper (55%) growth ceiling (Ridge et al., 2015), have proven to be more complex due to factors such as reef age, local tidal dynamics and environmental conditions (Bost et al., 2021). These general optimal growth zones and growth limitations are not solely determined by species‐specific ecological tolerances to emergence but are also influenced by population‐level thresholds, including factors such as larval recruitment abundances and survival rates, affected by predator pressure, food availability, competition for space and biofouling (Bishop & Peterson, 2006; Walles et al., 2016). The potential of natural intertidal oyster reef structures to keep up with and adapt to actual sea level rise is linked to the gradient between minimal growth rates at the upper growth ceiling and peak growth within the optimal growth zone (Ridge et al., 2017). As the reefs mature, the reef crests achieve a dynamic equilibrium in their position relative to the mean sea level (Ridge et al., 2017), with upper growth ceilings reaching 60%–70% exposure time and an optimal growth zone up to ~50% with vertical growth rates of 20–40 mm yr^−1^ (for *C. virginica*) (Bost et al., 2021). While vertical reef growth rates at the reef crests lie in the order of local sea level rise and are only limited by the exposure time (Ridge et al., 2017), an accelerated sea level rise exceeding minimum growth rates at the lower growth boundaries is projected to reduce the spatial extent of the lower habitat boundaries (Ridge et al., 2015).

Similar to *C. virginica*, the first studies on intertidal reefs of the Pacific oyster *Magallana gigas* (Thunberg, 1793 formerly *Crassostrea gigas*) in the Oosterschelde (southern Netherlands) showed long‐term average vertical accretion rates of 7.0–16.9 mm yr^−1^ over approximately 30 years exceeding local rates of sea level rise (Walles, Mann, et al., 2015). However, a first case study on an *M. gigas* reef in the German Wadden Sea using drone‐based photogrammetry suggested limited adaptive potential to accelerated sea level rise exceeding 3 mm yr^−1^, contrasting previous studies by indicating restricted vertical resilience due to minimal growth rates at the uppermost reef top and the absence of a detectable optimal growth zone (Hoffmann et al., 2023).

Due to the global degradation of native oyster populations over the last century due to overfishing and pollution (McAfee & Connell, 2021), the fast‐growing species *M. gigas* was introduced in coastal aquacultures worldwide since the mid‐19th century (Miossec et al., 2009). Subsequently, *M. gigas* has established self‐sustaining wild populations outside aquaculture facilities, in part due to misconceptions about temperature‐dependent reproductive conditions (Anglès d'Auriac et al., 2017; Faust et al., 2017; Moehler et al., 2011), raising concerns about its irreversible ecological impacts on native species and their associated ecosystems (Herbert et al., 2016; Miossec et al., 2009; Reddin et al., 2022).

General warming trends in coastal water masses are projected to expand *M. gigas* northern distribution and higher frequencies in spawning events, thereby increasing population resilience (King et al., 2021; Teixeira Alves et al., 2021). However, both native and non‐native oyster species worldwide are susceptible to extreme meteorological events (Cheng et al., 2016) and emerging diseases (Mazaleyrat et al., 2022), highlighting the vulnerability of coastal oyster populations.

*Magallana gigas* reef structures have been reported along the Atlantic coast from Spain to Norway, providing ecosystem services similar to those of native oyster reefs (Hansen et al., 2023). One prominent example is the trilateral (Denmark, Germany, Netherlands) Wadden Sea area, where mussel beds of the native blue mussel (*Mytilus edulis*) served as settling ground for *M. gigas* on the spacious tidal flats (Markert et al., 2010; Wehrmann et al., 2000).

Within the first decade after bioinvasion, former mussel beds were widely transformed into oyster reefs. However, *M. edulis* benefits from the reef structures as habitat (Reise, Buschbaum, Büttger, & Wegner, 2017). The protected status of the Wadden Sea enables *M. gigas* to exhibit their natural dynamics (Smaal et al., 2005) by forming diverse spatial patterns and internal reef zonation (Hitzegrad et al., 2022; Markert, 2020), redrawing previous mussel bed structures and locally covering up to 6% of the tidal flats (Folmer et al., 2017). Population dynamics and spatial distribution of *M. gigas* were extensively monitored during their invasion (Reise, Buschbaum, Büttger, Rick, et al., 2017) and are part of recent monitoring efforts (Folmer et al., 2017), revealing important factors that limit and facilitate successful reproduction, dispersal and growth. High water temperature and temperature dynamics during summer months are elementary for successful spawning events and optimal growth in the Wadden Sea (Diederich et al., 2005), whereas regional larval dispersal is mainly driven by tidal currents (Schmidt et al., 2008). *M. gigas* seems tolerant to cold winters (Nehls et al., 2006) and heat stress (Rahman et al., 2019), while ice drift can cause mechanical abrasion on the reefs (Diederich et al., 2005; Reise, Buschbaum, Büttger, & Wegner, 2017). Growth metabolism activity is a complex interplay mainly between temperature and food availability; under high food availability, growth is optimal at 20°C water temperature, sharply decreasing from 25 to 30°C (Kamermans & Saurel, 2022). Individuals can reach a maximum shell length of up to 300 mm, with an annual growth rate of 25–46 mm yr^−1^ (Diederich, 2006; Schmidt et al., 2008). *M. gigas* demonstrates a wide salinity tolerance range (10–42 PSU) (Mann et al., 1991), showing greater resilience to hypersaline waters (Nell & Holliday, 1988), while low salinities led to reduced growth and reproductive success (Wiltshire, 2007). Intensive growth experiments have disclosed an optimal growth zone within the tidal frame for *M. gigas*, analogous to those observed for *C. virginica* (Walles et al., 2016), with oysters exhibiting a general type III infant mortality during their life cycle by experiencing exponentially high mortality during the first 2 years (Walles, Mann, et al., 2015).

Despite the non‐native character of *M. gigas*, its establishment in European coastal waters offers unique insights into the long‐term effects of non‐native ecosystem engineering species and their adaptive responses to shifting environmental conditions. Hereby, they offer the potential to provide additional ecosystem services attributed to their complex biogenic structures, for example, increased frictional wave attenuation induced by the sharp ventral margins of the densely arranged shells (Borsje et al., 2011; Hitzegrad, Brohmann, et al., 2024; Hitzegrad, Köster, et al., 2024). Additionally, the biosedimentary reef facies fundamentally impact carbonate sedimentation in the Wadden Sea (Bungenstock et al., 2021), potentially creating future geo‐archives (Petersen et al., 2021). In the dynamic Wadden Sea ecosystem, the temporal stability and future persistence of these reefs remain largely unknown, particularly in determining if the reproductive output of existing populations is sufficient for their future persistence. Studies combining population dynamics and vertical accretion of *M. gigas* reefs also emphasize the importance of understanding underlying population dynamics that support reef accretion to fully leverage the potential usage of oyster reefs as a tool for coastal protection strategies (Walles et al., 2016; Walles, Mann, et al., 2015; Walles, Salvador de Paiva, et al., 2015), for example, supporting measures to stabilize coastlines and tidal flats and to mitigate hydrodynamic impacts. Therefore, it is essential to comprehend how oyster reef growth responds to varying population conditions and dynamics (Walles, Mann, et al., 2015), shaped by numerous biophysical factors and environmental stressors. As climate change and sea level rise alter hydro‐morphodynamic and biological conditions, this understanding will be crucial in assessing how oyster reefs will cope with changing environments.

This study aims to analyse seasonal vertical growth dynamics of two intertidal oyster reefs in the central Wadden Sea and their associated population dynamics. We hypothesize that variations in seasonal vertical reef growth will be connected to changes in population structure and individual growth. Elucidating this relationship will improve our comprehension of principal factors controlling oyster reef growth. Our specific objectives are: (1) to track seasonal vertical reef dynamics using terrestrial laser scanning; (2) to analyse growth patterns via seasonal data in population dynamics; (3) to relate vertical growth rates to population dynamics, assessing the impact of oyster population structure and dynamics on vertical reef growth and (4) to evaluate the resilience of these reefs against projected sea level rise.

## 2 MATERIALS AND METHODS

### 2.1 Study sites

**FIGURE 1:**
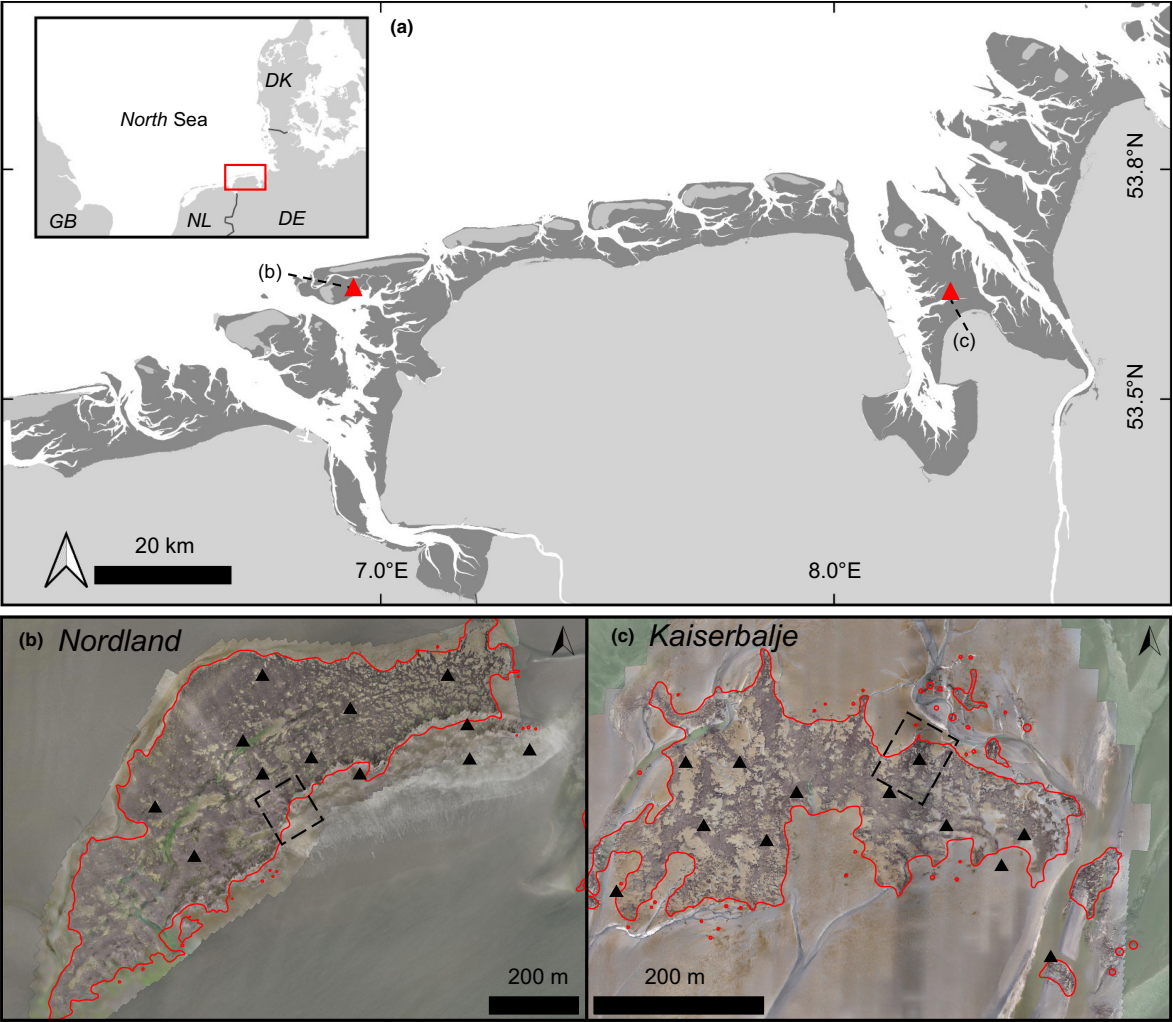
(a) Location of the study sites (red triangles) in the central Wadden Sea (intertidal areas in dark grey (Federal Waterways Engineering and Research Institute (BAW), 2024)). (b) Drone image mosaic of oyster reef Nordland (NL) (53.6424960° N, 008.9411970° E). (c) Drone image mosaic oyster reef Kaiserbalje (KB) (53.6470116° N, 008.2664760° E). Drone image mosaic of KB was modified after Hoffmann et al. (2023), mosaic of NL was generated with the same method. Reef area is outlined in red (Nationalparkverwaltung Niedersächsisches Wattenmeer, 2021). Black triangles: Biological sampling locations, dashed squares: Terrestrial laser scanning area. Map created in QGIS version 3.32.2.

This study focuses on two intertidal oyster reefs in the central Wadden Sea (Figure 1a), namely, the Kaiserbalje reef (KB; Figure 1a,c) and the Nordland reef (NL; Figure 1a,b). Fieldwork was conducted semi‐annually before and after the main growth season; population dynamics surveys spanned from autumn 2019 to spring 2022 and terrestrial laser scanning was undertaken from spring 2020 to spring 2022 at KB, and from autumn 2020 to spring 2022 at NL. The terrestrial laser scanning and population dynamics surveys were conducted within the same week, with the exception of 2022 at NL with a 2‐week discrepancy. Both study sites have been the subject of long‐term monitoring efforts (Markert, 2020; Markert et al., 2010; Schmidt et al., 2008). The KB reef, located between the Jade tidal channel and the Weser River Estuary, spans 4.2 ha in an area with a tidal range of around 3.37 m (Federal Maritime and Hydrographic Agency (BSH), 2023). The surrounding sedimentary environment transitions from a sandy tidal flat in the south to a mixed tidal flat in the north. The first oysters were detected in 2004 (Schmidt et al., 2008), and the reef contrasts against the surrounding mudflats with oysters primarily adopting a vertical orientation.

The NL reef, situated in the tidal flats south of the barrier island Juist, covers 22 ha with a tidal range of around 2.53 m (Federal Maritime and Hydrographic Agency (BSH), 2023). The sediment composition varies from fine sands with shell debris and shell lag deposits in the south to predominantly fine sand in the north. The oysters at NL, first recorded in 1998 (Wehrmann et al., 2000), exhibit a more irregular orientation.

### 2.2 Terrestrial laser scanning surveys

The spatial analysis focused on representative areas of around 80 × 80 m covering central and marginal reef parts; here, we define the central reef part according to the zonation approach for the inter‐reef structural diversity by Hitzegrad et al. (2022). Approximately 20 individual 3D laser scans per study site and survey were carried out within a single low tide period using two laser scanners of the FARO Focus3D models X130, S120 and Splus150 (FARO Technologies Inc., FL, USA). The scanners were used to achieve an accuracy of ±2 mm at a distance of 25 m (FARO Knowledge Base, 2023) and were configured to a point spacing of 3–6 mm at a 10‐m distance. A total of 106 scans at KB and 96 scans at NL were used to generate seasonal 3D models. To ensure accurate registration of individual scan point clouds during post‐processing, 15–17 movable reference spheres (Ø145 mm) were randomly placed in terrestrial laser scanning survey areas. In addition, 13–15 reference spheres were placed on permanently fixed iron rods (900 × 10 mm), deeply penetrated into the sediment and used to establish a local coordinate system throughout the entire study period. Their positions were determined using a Stonex‐9000‐dGPS (Real Time Kinematic and local geoid model GCG2016NW corrected), with a theoretical horizontal accuracy of 8 mm and a vertical accuracy of 15 mm. The surface reference for elevation heights is defined by the German standard datum (Normalhoehennull NHN) for mean sea level.

#### 2.2.1 Post‐processing of terrestrial laser scanning raw data

Initial processing of individual 3D scans was conducted in the FARO Scene software (FARO Technologies Inc., v.2019.01‐2023.01). The registration of single scan point clouds from each campaign resulted in a ScanScene. This was achieved using the positions of both movable and fixed reference spheres, with individual scan point clouds clipped to a maximum extent of 30 m. For co‐registration of the subsequent seasonal ScanScenes, the ScanScene of the first survey was designated as the Reference‐ScanScene, with subsequent ScanScenes spatially aligned based on all suitable fixed reference sphere positions. This method was preferred over pairwise co‐registration and the use of a reference subset to ensure overall optimal alignment. Global positioning of the survey areas was facilitated using dGPS‐coordinates (UTM Zone 32). Further post‐processing was conducted in the open‐source software CloudCompare (v.2.13) (Girardeau‐Montaut, 2022).

#### 2.2.2 Vertical reef growth

**FIGURE A1:**
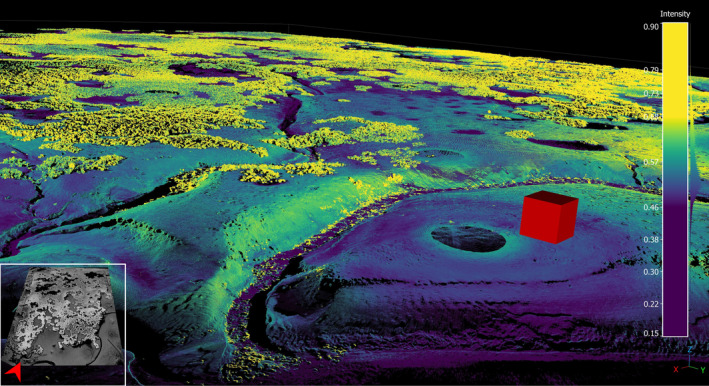
Point cloud perspective view of the terrestrial laser scanning area at the Kaiserbalje oyster reef. Intensity values are coloured and show the higher backscattered laser intensity on the oyster reef. Overview map in the left corner shows the viewpoint. Red scaling quadrat measures 1 × 1 × 1 m.

A region of interest (ROI) was defined as the oyster reef surface within the scanned area, excluding the surrounding and internal sediment surfaces. Large sediment areas were manually excluded. The reflected laser beam intensity (Figure A1) of individual scan point clouds, which exhibited higher reflectance on oysters than on surrounding sediment and metrics of local surface variation was calculated in the CloudCompare toolbox to compute statistical parameters on a scale of 30–100 mm, which were further used for segmentation. The resulting ROI was uniformly clipped across all ScanScenes (~ 65 × 80 m at KB, ~ 70 × 85 m at NL). The ROI point clouds were then used to conduct a pairwise calculation of vertical differences between ScanScenes using the Multiscale Model to Model Cloud Comparison (M3C2) algorithm (Lague et al., 2013). For each pair, the point cloud of the first ScanScene was subsampled to a resolution of 50 mm and employed as Corepoints for the M3C2 analysis. Vertical normals and a projection scale of 50 mm were employed to calculate vertical M3C2 distances. The projection scale defined the radius within neighbouring points of the Corepoints was selected for the computation of the mean distance. The median, interquartile range (IQR), skewness and kurtosis for Corepoints that included more than four points of each of the paired ScanScenes were determined from the M3C2‐Distance frequency distribution. To evaluate the relation between elevation and vertical reef dynamics, the M3C2‐distance frequency distribution was categorized into 20 mm elevation bins (Bost et al., 2021) from the first ScanScene of each pairwise comparison. For each elevation bin, the median vertical distance was determined with a 95% confidence interval and an optimal growth zone was assessed based on the highest median growth values according to Bost et al. (2021).

The general assessment of relative elevation‐related exposure time in the intertidal regime is based on three different methods: (i) discrete measurements of aerial exposure from nearby tide gauges (Ridge et al., 2017) or in situ pressure loggers (Bost et al., 2021); (ii) calculations based on the local mean tidal range, from mean low water to mean high water (MTR; 6 h of exposure equating to 50%) (Walles, 2015) and (iii) assessments based on a linear tidal curve (Hoffmann et al., 2023). We estimated the percentage aerial exposure in relation to elevation positions by (i) using 15‐min water level data from nearby tide gauges. For KB data were queried from the Hooksielplate tide gauge (ID: 9430020) 8 km northwest of the reef and for NL from the Norderney Riffgat tide gauge (ID: 9360010) 15 km east of the reef (Federal Waterways and Shipping Administration (WSV), 2023a), from the first to the last terrestrial laser scanning survey week (in the following measured exposure time as % MET). To evaluate seasonal fluctuations in elevation‐related exposure time and in mean water level, tide gauge data from individual seasonal scan intervals were used. Aerial exposure time was also estimated by (ii) using mean low water to mean high water level in a 745‐min sinusoidal tidal cycle (KB: −1.76 to 1.61 m NHN; NL: −1.29 to 1.24 m NHN (Federal Maritime and Hydrographic Agency (BSH), 2023)) (in the following % MTR).

#### 2.2.3 Uncertainty estimation

Given the highly dynamic nature of the study area, no objects are considered completely stable, as commonly applied in other studies for alignment error estimation (Anders et al., 2020; Esposito et al., 2017; Jackson‐Bué et al., 2021). The iron rods, while potentially affected by factors such as ice drift (rare) (Federal Maritime and Hydrographic Agency (BSH), 2024b) and storms (common) (German Meteorological Service (DWD), 2024), reflect the most stable elements in the scan area. Potential misplacement (<5 mm) of the reference spheres on the iron rods from the local coordinate system introduces further uncertainties. To evaluate the accuracy of the local coordinate system, 3D distances between reference sphere positions within seasonal ScanScenes were compared across all ScanScenes. Over shorter distances, such as those within the used range of individual laser scans (30 m), residuals between ScanScenes are expected to be primarily influenced by potential movement of the iron rods, sphere misplacement and scanner uncertainties rather than registration uncertainties. For larger distances, residuals are more influenced by registration uncertainties and sphere misplacement, as distances are calculated from cascading spatially aligned individual scans.

For KB, the distances ranged from 15.6 m to 113 m. On distances <30 m, the mean residual of distances was 7.97 mm, and for all distances, the mean residual of distances was 10.81 mm. For NL, distances ranged from 14.5 m to 118.9 m, with mean residuals for distances <30 m of 3.94 mm and for all distances 6.36 mm. To estimate the overall alignment uncertainties, we calculated the 3D distances between the mean positions of each fixed reference sphere centroid of each ScanScene to its correspondent in all other ScanScenes. From these distances, the vertical, lateral and 3D root mean square (RMS) were derived. At KB, 12 reference positions were used for alignment throughout the study period. At NL, for October 2020 only four reference positions were available, for further three ScanScenes 13 reference positions were available. The uncertainty estimation, based on the overall 3D RMS between fixed reference positions, was 17 mm for KB and 15 mm for NL. The lateral RMS was 15 mm for KB and 14 mm for NL, while the vertical RMS was 8 mm for KB and 5 mm for NL (for RMS of ScanScene pairs refer to Table A1). Given that the lateral and 3D RMS is below half of the M3C2 projection scale of 50 mm (except for the two comparisons containing NL September 2020 with a lateral RMS of 26 mm and 27 mm), it can be assumed that corresponding points are recognized for the calculation of vertical M3C2 distances. Therefore, the vertical RMS is considered to provide a reliable estimate of the overarching vertical uncertainty within the ROI. Furthermore, the FARO Scene software computes an internal, not fully evaluable, reference sphere tension for the co‐registration of 8.95 mm for KB and 9.37 mm for NL, with reference sphere mean distance, horizontal and vertical errors of 7.9 mm, 6.4 mm and 3.4 mm for KB and 7.5 mm, 6.4 mm and 3.0 mm for NL. For visual validation, we inspected both raw and ROI point clouds for alignment quality and investigated regional erosion, sedimentation, tidal creek dynamics and reef subsidence phenomena using CloudCompare.

### 2.3 Population dynamics

To analyse the interaction of population dynamics and reef growth, 12 biological samples were collected semi‐annually (i.e. spring and autumn), randomly distributed across each reef (Figure 1b,c). Sampling was carried out using a set frame size of 25 × 25 cm (0.0625 m^2^) by collecting all oysters (individuals and disarticulated shells) from the taphonomic active zone (approx. topmost 25 cm of the reef surface). Sample processing included measuring the shell length (SL) of live and dead individuals of *M. gigas* at the longest point from the hinge to the posterior margin with a calliper to the nearest millimetre. Adult individuals >25 mm in SL were categorized into size classes with intervals of 25 mm, representing average annual growth. Ten cleaned individuals per size class were measured for the parameters live wet weight (LWW), SL, shell weight (SW) and weight of cooked flesh (WCF) according to a trilateral standard (Nehls et al., 2009) to ensure comparability of data with previous monitoring programmes (Markert, 2020). Population data of both reefs from 2006 to 2013 were integrated for long‐term trends in reef development (Markert, 2020). From this, we calculated: (i) the average LWW m^−2^ for each sampling season, determined using a non‐linear logistic regression that correlates LWW with SL, (ii) the condition index (CI) for each season, according to Davenport and Chen (1987), computed with the following equation:

$$\mathrm{CI}=100\times \frac{\mathrm{WCF}}{\mathrm{WCF}+\mathrm{SW}}.$$

To assess the growth and related abundance of individual oyster cohorts, we determined length‐frequency distributions (LFD) for each reef and survey. Lengths were converted into mean abundances m^−2^ and categorized into 5 mm bins. Initial age estimations were deduced based on the knowledge of annual growth rates (Schmidt et al., 2008; Walles, Mann, et al., 2015). Length cohorts were first identified by decomposing LFDs into multiple Gaussian distributions using the Bhattacharya method integrated within the FISAT II software (Gayanilo et al., 2005). Initial estimations were improved using the Gaussian mixture model of the R package *AdaptGauss* (Ultsch et al., 2015) via R (v4.2.1) using RStudio (v022.07.2 + 576). To assess seasonal growth patterns during winter and summer and to evaluate the occurrence of spawning events, for which water temperature is the principal driver, we had to rely on external data due to the absence of on‐site data loggers for physical parameters during our study period 2019–2022. We obtained mean daily water temperatures from local stations, Dwarsgat, Germany, situated 5 km northwest of KB (Federal Waterways and Shipping Administration (WSV), 2023b), and Borkum Fischerbalje, Germany, 15 km southwest of NL (Federal Maritime and Hydrographic Agency (BSH), 2024a). Missing data were supplemented by local sea surface temperature data (ESA Climate Change Initiative and the SST CCI project (Merchant et al., 2019; Merchant & Embury, 2020)). From this data, we calculated the number of days with temperatures below 5°C, indicative of increasing cold stress (Zhu et al., 2016), and above 10°C, which is considered to be a general threshold for enhanced shell growth in *M. gigas* (Coleman, 1986; Quayle, 1988) and *C. virginica* (Goodwin et al., 2021; Kirby et al., 1998). We also identified longer periods of consecutive days (*n* > 28) with temperatures exceeding 19.5°C, which was recognized as the threshold for intensified spawning (Cadée, 2001), similar to 19.7°C used for reproduction modelling (Teixeira Alves et al., 2021).

### 2.4 Linking vertical reef growth to population dynamics

To compare seasonal sampling intervals, we divided vertical reef growth rates and oyster size increments by the time between field campaigns, assuming that both methods (terrestrial laser scanning and population dynamics) cover average vertical reef growth and population dynamics. We correlated individual growth with the relative number of days exceeding 10°C, predicting that increased growth rates would reflect warmer periods and thus allow for a general separation between winter and summer intervals. Besides direct comparison of vertical reef growth and oyster size increments, we analysed the impact of living oyster abundance combined with length increment, applying a square root transformation to length ranges (>25, >50 and >75 mm) of the initial population, which predominantly dictates the short‐term vertical reef growth potential. This analysis acknowledges that larger oysters disproportionately influence reef dynamics due to their greater volume and surface area (Walles, Mann, et al., 2015). Linear ordinary least squares (OLS) regressions were computed using the software PAST: Palaeontological statistics software package for education and data analysis (v.4.16) (Hammer et al., 2001).

## 3 RESULTS

### 3.1 Vertical reef growth by terrestrial laser scanning

**FIGURE 2:**
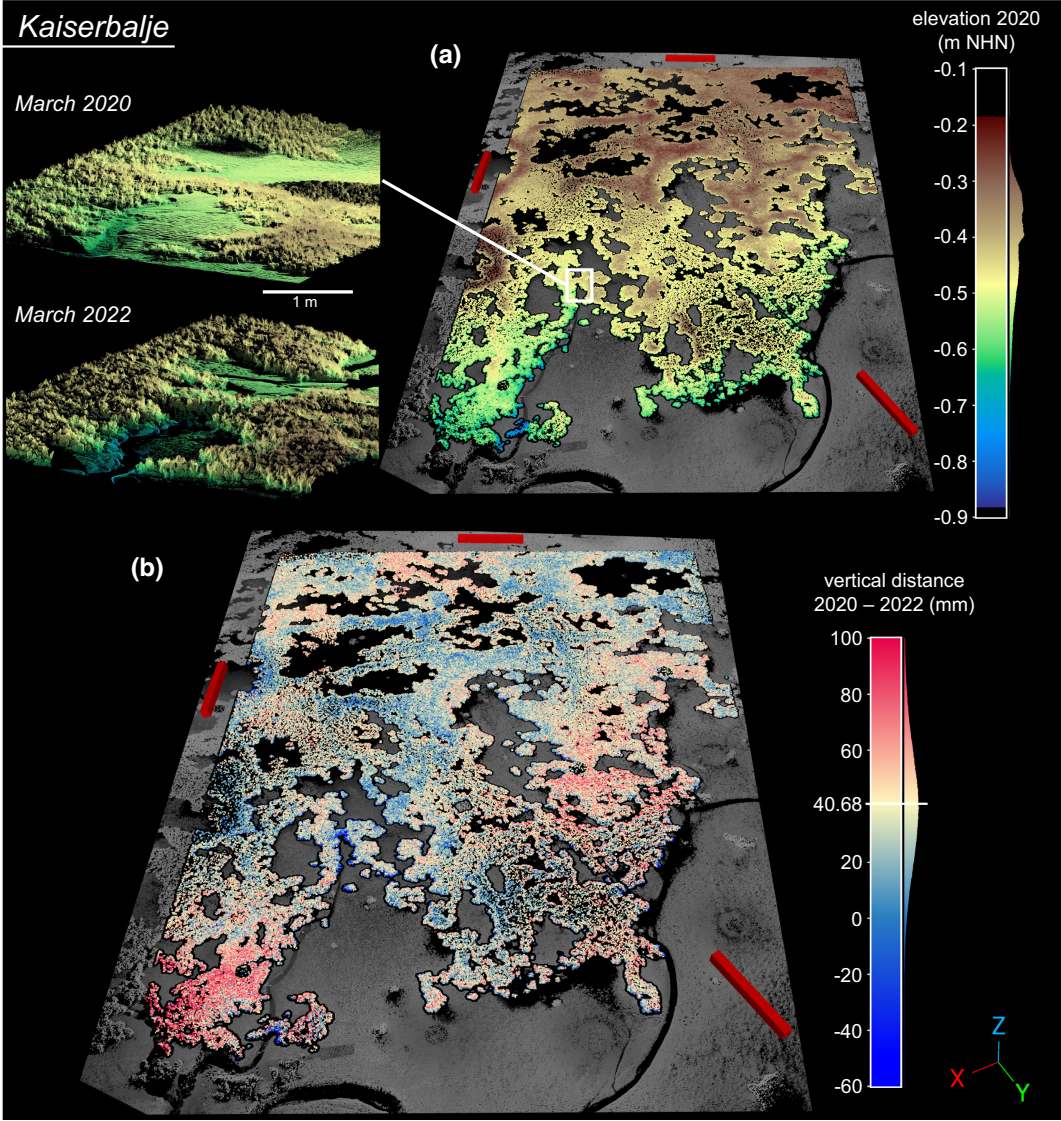
Analysis of elevation and vertical reef growth at the Kaiserbalje reef. Red scale bars indicating 10 × 1 × 1 m, y‐axis indicating north direction. (a) Elevation model of the Kaiserbalje ROI in March 2020. The detailed view displays reef internal sediment dynamics and reef internal drainage. (b) Visualization of M3C2 vertical distance between March 2020 and March 2022. Median vertical distance is added and centres the divergent colour scale.

**FIGURE 3:**
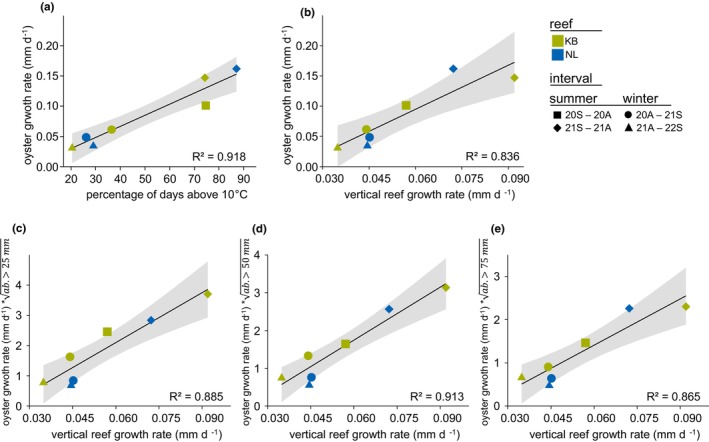
Correlation of biotic and abiotic factors in oyster and reef growth rates, respectively, for the reefs Kaiserbalje (KB) and Nordland (NL). (a) Correlation of daily oyster growth rates with temperature (percentage of days above 10°C), based on the combined mean growth rate data from the 2018 and 2019 cohorts. (b) Correlation between daily vertical reef growth rates and daily oyster growth rates from seasonal samplings. Correlation of daily vertical reef growth rate with the product of daily oyster growth rate and square root transformed abundance of oysters (c) >25 mm, (d) >50 mm and (e) >75 mm.

For KB (cp. Figure 2), the vertical M3C2 frequency distribution from the initial to the final terrestrial laser scanning survey (March 2020 to March 2022) revealed a positive median of 40.68 mm (IQR: 25.87–56.03 mm) with a sum of seasonal median vertical distances of 44.05 mm (10.78 + 8.33 + 19.33 + 5.61 mm). The seasonal comparison of ScanScenes showed higher daily vertical growth rates during summer compared to winter periods (Figure 3). The highest vertical change was observed from March to October 2021 with a median of 19.33 mm (IQR: 9.81–29.22 mm), corresponding to 0.09 mm d^−1^. A lower rate (0.06 mm d^−1^) was observed from March to September 2020 with a median of 10.78 mm (IQR: 0.81–21.83 mm), with a positive asymmetric distribution (skewness of 0.76).

**FIGURE 4:**
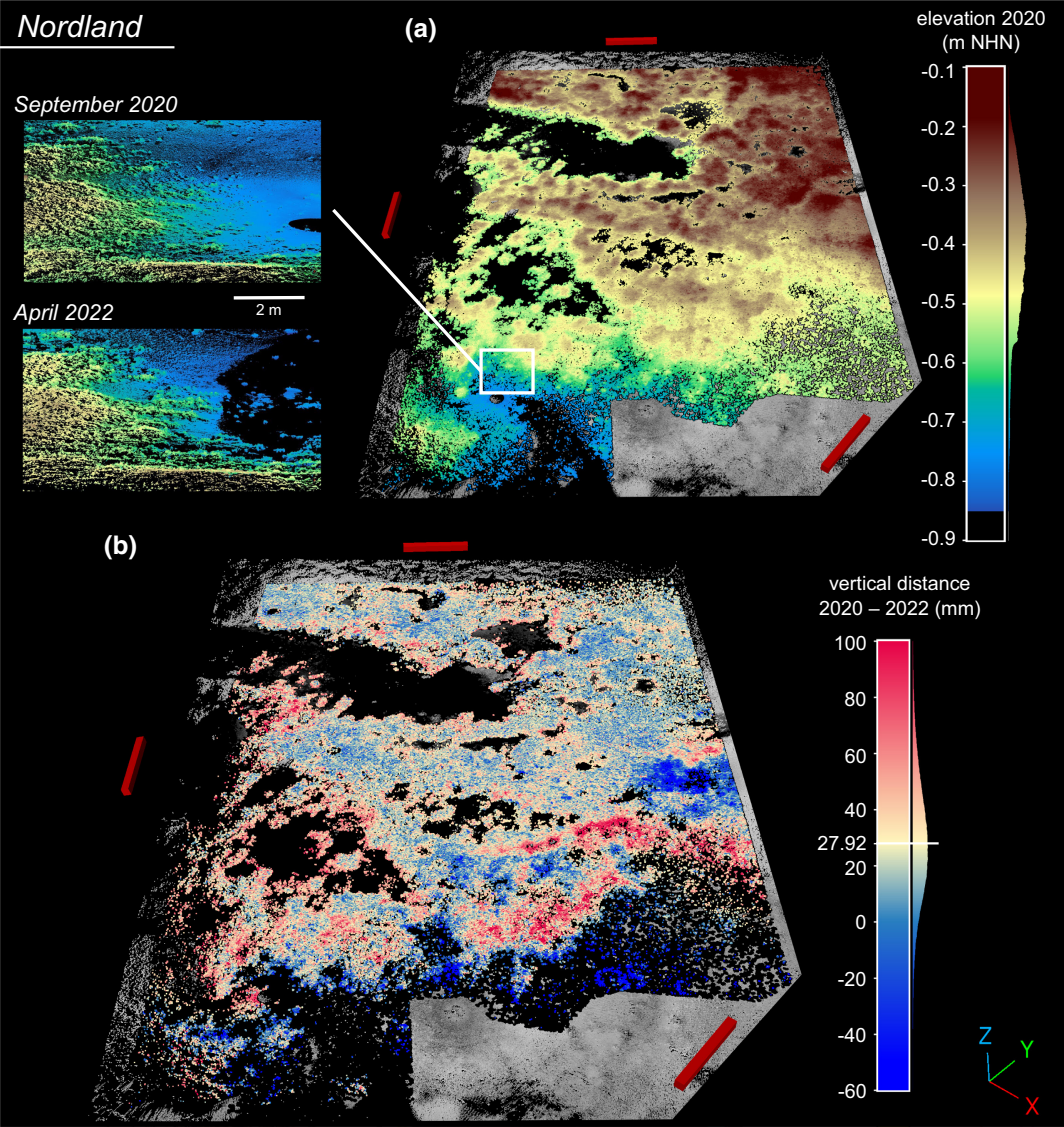
Analysis of elevation and vertical reef growth at the Nordland reef. Red scale bars indicating 10 × 1 × 1 m, y‐axis indicating north direction. (a) Elevation model of the Nordland ROI in September 2020 (a). The detailed view displays dynamics at the reef edge. (b) Visualization of M3C2 vertical distance between September 2020 and April 2022. Median vertical distance is added and centres the divergent colour scale.

At NL (cp. Figure 4), the vertical M3C2 frequency distribution from September 2020 to April 2022 revealed a positive median of 27.92 mm (IQR: 13.83–43.50 mm), with a sum of semi‐annual median vertical distances of 30.05 mm (9.48 + 10.62 + 9.95 mm). During the summer period of 2021 (April to September), a higher daily vertical growth rate (0.07 mm d^−1^) was observed, exceeding the rates during the preceding and following winter periods (0.05 and 0.04 mm d^−1^).

**FIGURE 5:**
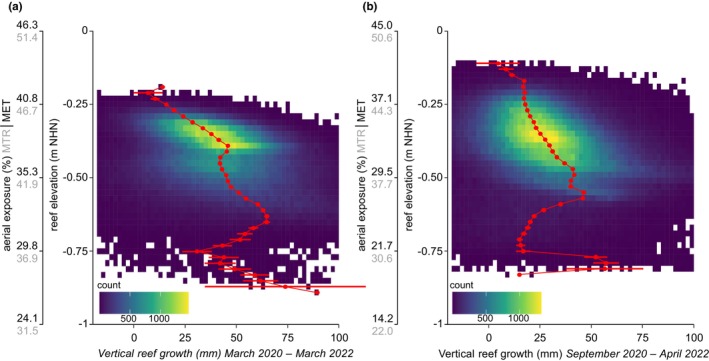
Analysis of oyster reef growth across elevation and aerial exposure gradients for (a) Kaiserbalje (KB) and (b) Nordland (NL). The median vertical growth for 20‐mm elevation bins is represented by red dots, with whiskers denoting the 95% confidence interval. The colour gradient illustrates the combined density for each 20‐mm elevation bin and a 2‐mm vertical reef growth bin. Aerial exposure time is given in per cent in relation to data from nearby tide gauges as measured exposure time (MET) and using mean low water to mean high water level (MTR).

At the beginning of the study, the ROI elevation from lowest to highest calculated 20 mm bin mid‐point for KB (March 2020) ranged from −0.89 m to −0.19 m NHN, and for NL (September 2020) from −0.83 m to −0.09 m NHN. The combined density of elevation and vertical growth rates from the initial to the final observation reveals the uneven distribution of reef area across the elevation gradient (Figure 5), attributed to the reef's morphology and the ROI setting. Whereby 90% of the ROI Corepoints for KB were situated between −0.59 m and −0.30 m NHN, and for NL between −0.58 m and −0.24 m NHN. The mean height of the ROI was similar at both sites (KB −0.42 m ± 0.09 m NHN; NL −0.39 m ± 0.10 m NHN), whereas the percentage of aerial exposure time differs based on the local tidal range. For KB, it was 43.4% MTR and 37.1% MET; for NL, 40.7% MTR and 32.8% MET.

**FIGURE A2:**
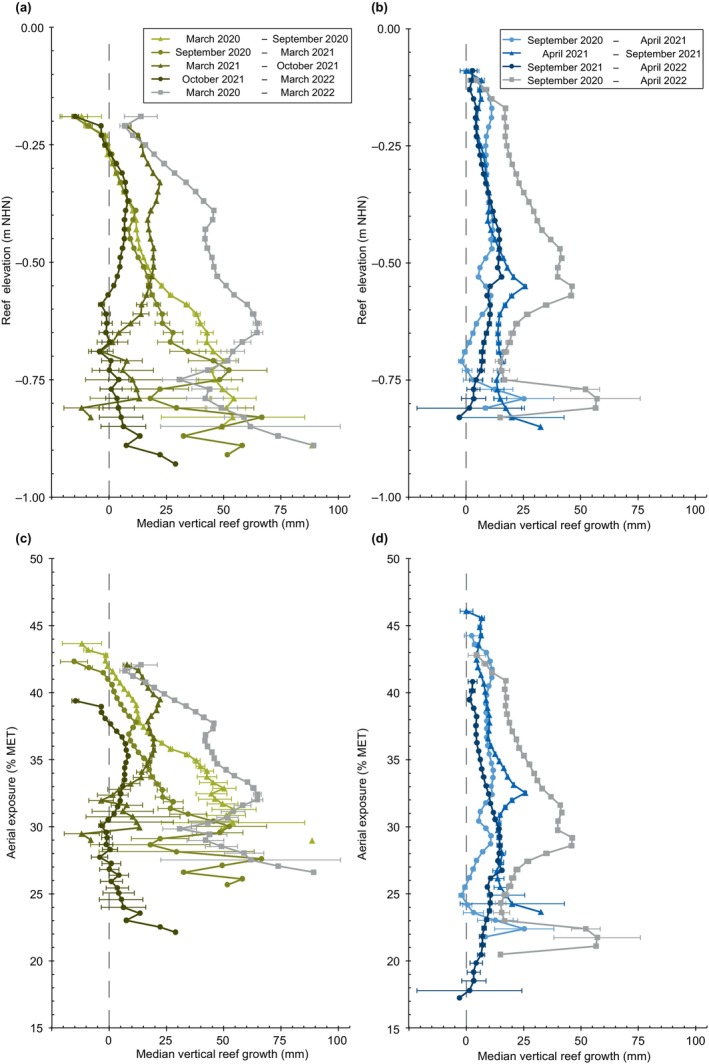
Median vertical oyster reef growth across elevation gradients for Kaiserbalje (a) and Nordland (b). Median vertical oyster reef growth across aerial exposure gradients for Kaiserbalje (c) and Nordland (d). Aerial exposure time is given in per cent in relation to seasonal data from nearby tide gauges as measured exposure time (MET). The median vertical growth for each 20 mm elevation bin is represented by symbols, with whiskers denoting the 95% confidence interval.

For KB, the optimal growth zone ranged from −0.56 m NHN to −0.68 m NHN, peaking between −0.62 m NHN and −0.66 m NHN with a maximum median growth of 64.64–64.72 mm (in 25 months). Two elevation‐related vertical growth gradients were identified for KB: one superimposed from the higher reef crest to the lower marginal areas and a second internal gradient inside the fringing reef structure from the smaller ridges to their lower margins (Figure 2). Low vertical growth values below −0.68 m NHN were located at the lower reef edges (Figure 2). For NL, the optimal growth zone ranged from −0.46 m NHN to −0.58 m NHN, peaking between −0.54 m and −0.58 m NHN with a maximum median growth of 45.85–46.25 mm (in 19 months). With decreasing elevations, vertical M3C2 values initially decreased and then increased again in the transition region, where the reef structure is characterized by individual oyster clusters (Figure 4). Furthermore, seasonal elevation‐related variations in vertical growth were observed for KB (Figure A2a), with increasing M3C2 values towards lower edges of the reef structure in the first two scan intervals (March to September 2020 and September 2020 to March 2021). In contrast, the following two scan intervals (March 2021 to October 2021 and October 2021 to March 2022) showed higher M3C2 values at higher locations, decreasing towards lower lying areas. NL displayed more consistent distributions in seasonal comparison (Figure A2b). Water levels in the KB and NL areas, as estimated by seasonal tide gauge data, exhibited fluctuations over the respective scan intervals (Table A1). In the KB area, the mean water level was observed to be 0.07 ± 1.20 m NHN from March 2020 to March 2022. During the final interval, from October 2021 to March 2022, an exceptionally high mean water level of 0.20 ± 1.21 m NHN was recorded. In the NL area, the mean water level was observed to be 0.05 ± 0.93 m NHN from September 2020 to April 2022. The final scan interval, spanning from September 2021 to April 2022, also exhibited the highest mean water level, reaching 0.18 ± 0.90 m NHN. The seasonal fluctuations in water level resulted in alterations to the specific elevation‐related exposure time for each scan interval (Figure A2c,d). By accounting for the respective exposure time at each elevation, the exposure time‐dependent seasonal M3C2 values revealed that the areas of highest growth at KB overlap at values of 30–40% exposure time.

### 3.2 Population dynamics

**FIGURE 6:**
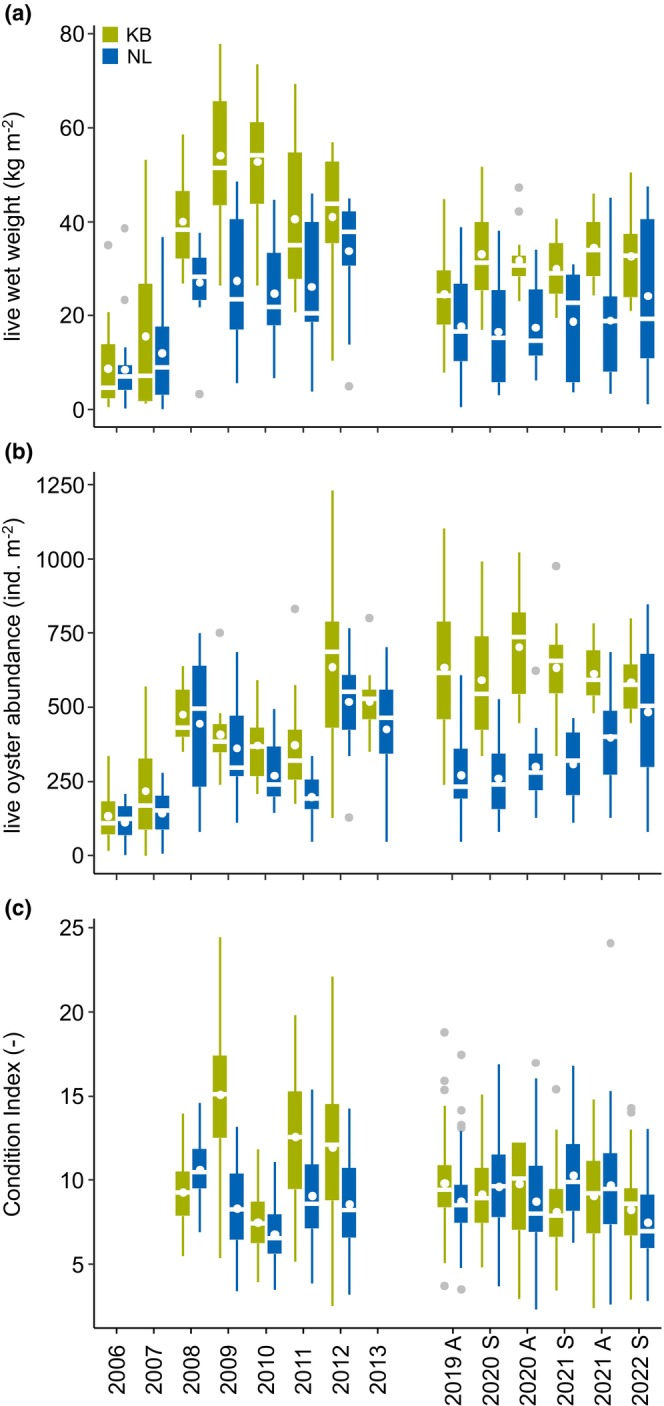
Live wet weight (a) and abundance >25 mm shell length (SL) (b) per square metre, and the condition index (ci) (c) of *M. gigas* for Kaiserbalje (KB) (green) and Nordland (NL) (blue) from autumn 2019 (a) to spring 2022 (S), with data from prior reef monitoring 2006–2013. The box plots illustrate the median as the horizontal line inside the box, the mean indicated by the dot within the box, the 25th and 75th percentiles defined by the box boundaries, the 10th and 90th percentiles denoted by the whiskers, and outliers shown as grey dots.

**FIGURE 7:**
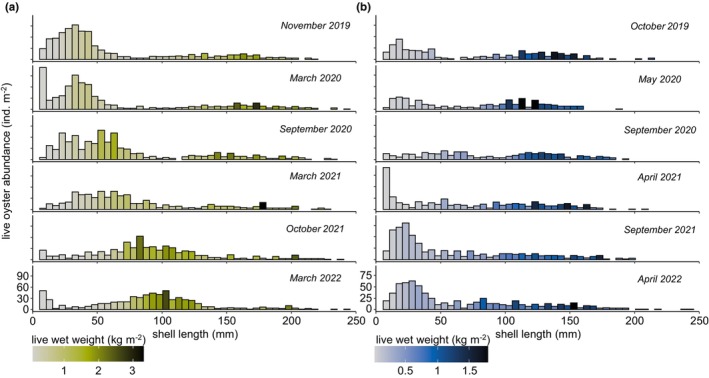
Length frequency distribution of *M. gigas* from autumn 2019 to spring 2022 at the oyster reef locations: Kaiserbalje (KB) (a) and Nordland (NL) (b). The live oyster abundance depicts the mean abundance of individuals per square metre, categorized in 5‐mm length bins. Colour gradients indicate the live wet weight of each corresponding 5 mm bin.

At both studied oyster reefs, the CI was higher in autumn compared to previous spring, ranging overall between (Mean ± S.D.) 7.5 ± 2.5 and 10.3 ± 2.7 with no pronounced variation throughout the study period (Figure 6c). The overall biomass as LWW kg m^−2^ (Mean ± S.D. of means) from 2019 to 2022 was higher at KB (31.1 ± 3.5 kg m^−2^) than at NL (19.0 ± 2.7 kg m^−2^) (Figure 6a). No consistent seasonal trend was observed. For KB, the seasonal means in LWW kg m^−2^ from this study were consequently lower than the highest means documented in 2009 and 2010 (Figure 6a). For NL, they were lower than in 2012 and 2013. The mean abundance of living adult oysters (SL > 25 mm) from 2019 to 2022 was higher at KB (627 ± 43 ind. m^−2^) compared to NL (338 ± 87 ind. m^−2^) (Figure 6b). NL shows a continuous increase of living adult oysters from 2019 to 2022, which is not seen at KB. The highest decline in *M. gigas* population was recorded at KB during the winter of 2020–2021, leading to a 20% decrease in overall oyster abundance (Figure 7). The median abundance of living adult oysters at KB and NL from 2006 to 2013 followed similar trends of increasing and decreasing values, not seen in the study period from 2019 to 2022 (Figure 6b).

Analysis of the length frequency distribution for KB reveals two dominant cohorts of spatfalls from 2018 and 2019, which were traceable throughout the study period (Figure 7). Additionally, a minor spatfall in 2021 was detectable in autumn 2021 and spring 2022. At the start of the survey in autumn 2019, the abundance of cohorts older than 2018 (>61 mm) was markedly low (34%). At NL, oyster recruitment in 2018 and 2019 was lower than at KB, and strong recruitment occurred in 2020. At KB, fast‐growing oysters of the 2019 cohort could be distinguished in autumn 2019 but merged with the 2018 cohort by 2020. Furthermore, individual cohorts showed an increasing overlap at lengths >50 mm, with age‐specific separation of cohorts not being feasible for older cohorts than from 2018.

### 3.3 Linking vertical reef growth to population dynamics

The association between vertical reef growth and oyster population dynamics was assessed over seven survey intervals. Given the sample size (*n* = 7), the findings are deemed indicative, study‐specific trends rather than metrics with universal applicability. The percentage of days with water temperatures exceeding 10°C was utilized as a proxy to approximate oyster growth, with higher rates typically occurring during summer as opposed to winter periods. This metric allowed for the differentiation of growth patterns between winter and summer periods across both reefs (Figure 3a). Oyster growth rates (mm d^−1^) indicated a strong relationship with the percentage of days above 10°C, as evidenced by Pearson's coefficient squared of *R* ^2^ = .918 and described with: $\text{oyster growth rate}\;\left(\mathrm{mm}\;d^{-1}\right)=0.001837\times \%\text{days exceeding}\;10^{{}^{\circ}}C-0.007197$. Separation of oyster growth differences and % days exceeding 10°C in winter intervals were consistent. Differences between the summer seasons of 2020 and 2021 at KB were inconsistent with % days exceeding 10°C. Lowest and highest oyster growth rates corresponded to lowest and highest % days exceeding 10°C, with 0.03 mm d^−1^ observed at KB (October 2021–March 2022) with 20.5% of days exceeding 10°C, and 0.16 mm d^−1^ observed at NL (April 2021–September 2021) with 87.1% of days exceeding 10°C. Oyster growth rates mirrored the median vertical reef growth rates detected by terrestrial laser scanning (Figure 3b) with *R* ^2^ = .836 and described with: $\text{oyster growth rate}\;\left(\mathrm{mm}\;d^{-1}\right)=24.313\times \text{median vertical reef growth}\;\left(\mathrm{mm}\;d^{-1}\right)-0.051228$.

The summer season 2021 presented variable vertical growth rates between KB and NL, with NL displaying an oyster growth rate above the 95% confidence band proposed by the OLS regression in comparison to the median vertical reef growth rate. Population assessment in spring 2021 indicates a lower mean oyster abundance at NL with 399 ind. m^−2^ than at KB with 633 ind. m^−2^ (Figures 6b and 7). When factoring in spatial oyster abundance, the correlation between individual oyster growth rates and vertical reef growth was enhanced (Figure 3c–e), for oyster abundance >25 mm SL with *R* ^2^ = .885, for oyster abundance >50 mm SL with *R* ^2^ = .913 and for oyster abundance >75 mm SL with *R* ^2^ = .865. The linear regression lines were described by the following equations.

For oyster abundance >25 mm with:

$$\text{oyster growth rate}\;\left(\mathrm{mm}\;d^{-1}\right)\times \sqrt{\text{oyster abundance}\;\mathrm{ind}.m^{-2}>25\;\mathrm{mm}}=54.913\times \text{median vertical reef growth}\;\left(\mathrm{mm}\;d^{-1}\right)-1.1993$$

For oyster abundance >50 mm with:

$$\text{oyster growth rate}\;\left(\mathrm{mm}\;d^{-1}\right)\times \sqrt{\text{oyster abundance}\;\mathrm{ind}.m^{-2}>50\;\mathrm{mm}}=46.732\times \text{median vertical reef growth}\;\left(\mathrm{mm}\;d^{-1}\right)-1.0595$$

For oyster abundance >75 mm with:

$$\text{oyster growth rate}\;\left(\mathrm{mm}\;d^{-1}\right)\times \sqrt{\text{oyster abundance}\;\mathrm{ind}.m^{-2}>75\;\mathrm{mm}}=35.698\times \text{median vertical reef growth}\;\left(\mathrm{mm}\;d^{-1}\right)-0.73879$$

## 4 DISCUSSION

### 4.1 Vertical reef growth

Our study of seasonal population dynamics and vertical reef growth via terrestrial laser scanning reveals synchrony between the biological growth of individual oyster cohorts, spatial abundance and the resulting vertical reef growth (Figure 3). Our results of median vertical reef growth rates from KB (19.8 mm yr^−1^) and NL (17.5 mm y^−1^) are comparable with other studies on intertidal oyster reefs. *M. gigas* reefs in the Oosterschelde showed long‐time accretion rates from 7 to 16.9 mm yr^−1^, estimated based on depth profiles from around 30 years (Walles, Mann, et al., 2015), whereas constructed *C. virginica* reefs at the US East Coast show core‐based accretion rates of 27 ± 7 mm yr^−1^ (Rodriguez et al., 2014). Both average long‐term accretion rates probably include a deceleration in vertical growth as the reefs extend beyond their optimal growth zone over time into areas of lower growth potential and distinct phases of rapid and slower vertical propagation. Our estimated median vertical growth rates are strongly influenced by the proportional distribution of the specific ROI settings in terms of elevation (aerial exposure time) and the resulting differences in general growth potential.

The highest ROI elevations marking the reef crest at KB are at 47.8% MTR and 42.1% MET. For NL, the highest ROI elevations were at 48.4% MTR and 42.2% MET (Figure 5), which is just below the upper limit of reef occurrence described with ~50% MTR for *M. gigas* reefs in the Dutch Wadden Sea and ~55% for reefs in the Oosterschelde in 2011 (Walles, Salvador de Paiva, et al., 2015).

The optimal growth zone with highest vertical reef growth is at 39.1% MTR and 32.2% MET at KB and at 36.1% MTR and 27.7% MET at NL which fit within a general optimal growth zone of 20%–40% exposure time for *M. gigas* (Walles et al., 2016) and *C. virginica* reefs (Ridge et al., 2017). Furthermore, the peak growth rates at KB (31.5 mm yr^−1^) and NL (29.1 mm yr^−1^) are comparable to decade‐old constructed (27 mm yr^−1^) and natural (24 mm yr^−1^) *C. virginica* reefs (Bost et al., 2021) and to young constructed (few years) and centennial old natural reefs with peak growth rates of 28.9–66.7 mm yr^−1^ (Ridge et al., 2017).

Most recent investigations showed that the optimal growth zone can reach positions >50% aerial exposure for *C. virginica* reefs older than 50 years and an uppermost reef crest limit at ~60%–70% aerial exposure (Bost et al., 2021). In contrast, upper reef growth is limited to ~55% exposure time for reefs in the Oosterschelde, where low individual survival rates above this limit probably do not allow for new reef formation at higher locations (Walles et al., 2016). With oyster recruitment detectable up to 66% aerial exposure time (Walles et al., 2016), the relatively young North Sea reefs might also show maximal reef crest heights >50%–55% aerial exposure time over longer periods. Nevertheless, contrasting tidal ranges and dynamics between the Wadden Sea (meso‐ to macrotidal) and US East Coast studies (mainly microtidal) imply different optimal growth zones and growth ceilings (Bost et al., 2021) besides species‐specific ecological tolerances.

### 4.2 Controls of population dynamics on vertical reef growth

When interpreting vertical oyster reef growth, it is imperative to consider the population dynamics of the reef‐building oysters. With our results, we complement previous studies on reef accretion through shell accumulation (Walles, Mann, et al., 2015) and linkages between adult oyster density (abundance) and accretion rates (Ridge et al., 2015), which indicate the influence of population dynamics on reef growth and accretion through cumulated effects of oyster recruitment, growth and survival rates. As the oyster population demography, for example, the LFD at the beginning of a survey, fundamentally influences the spatial growth data obtained via remote sensing like terrestrial laser scanning, we want to emphasize the need to evaluate the preceding population and reef development that shapes these starting conditions; for both investigated oyster reefs, considering of long‐term monitoring data provides an important contribution to this assessment.

**FIGURE 8:**
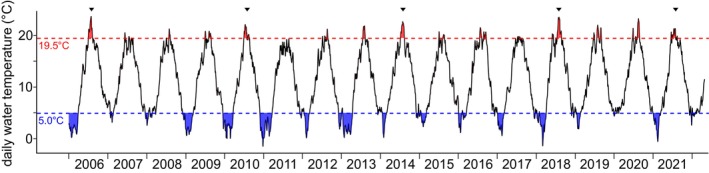
Daily mean water temperature in the Kaiserbalje area from 2006 to April 2022. The red line indicates temperatures exceeding 19.5°C, while the blue line represents temperatures below 5°C. Years marked by highest consecutive days (*n* > 28) exceeding 19.5°C are 2006 (*n* = 39), 2010 (*n* = 29), 2014 (*n* = 35), 2018 (*n* = 40) and 2021 (*n* = 34).

For *M. gigas* in the Wadden Sea, major spawning events were the primary driver of the exponential invasion phase, leading to the rapid transformation of mussel beds into oyster reefs and their subsequent establishment (Reise, Buschbaum, Büttger, & Wegner, 2017). One such event occurred in 2006, triggered by a positive water temperature anomaly (Figure 8, and Reise, Buschbaum, Büttger, Rick, et al. (2017)), leading to a peak in the abundance of adult oysters at KB in 2008, with biomass m^−2^ reaching its highest levels in 2009 and 2010 (Figure 6). Consecutively, one of the highest numbers of continuous days surpassing 19.5°C water temperature (Figure 8) was recorded during the European marine heatwave in the summer of 2018 (Kaiser et al., 2023). These exceptionally warm and persistent temperatures, which led to a large spatfall, were followed by two mild winters (Figure 8), resulting in a high abundance of the 2018 cohort at KB. Furthermore, the low number of larger oysters at KB and NL can be linked to unfavourable low‐temperature conditions for spawning in previous years (Figure 8), which aligns with observations in the Dutch Wadden Sea 2017–2019 (Ricklefs et al., 2017).

At NL, recruitment of the 2018 cohort was lower than at KB, and strong recruitment occurred in 2020, indicating variations in local conditions that influenced recruitment patterns. Nevertheless, populations of *M. gigas* on both reefs suggest a sustained state of good health, as indicated by natural seasonal variations of the CI (Brown & Hartwick, 1988; Costil et al., 2005).

**FIGURE A3:**
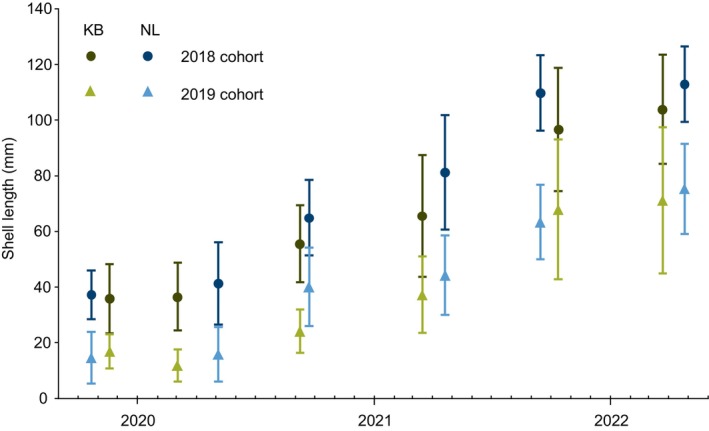
Mean oyster size over the study period by cohorts. Circles indicate the 2018 cohorts for Kaiserbalje (KB) and Nordland (NL), while triangles indicate the 2019 cohorts. Whiskers indicate ±1 SD.

Lower values in LWW kg m^−2^ from 2019 to 2022 compared to the early years of reef formation and decoupled trends in adult oyster abundances reflect the content of large (old) oysters as evidence for established population structures (Reise, Buschbaum, Büttger, Rick, & Wegner, 2017; Smaal et al., 2009), comparable to known temporal stability of restored *C. virginica* reefs (Smith et al., 2022). Inter‐annual variations in vertical reef growth, as observed in our study and others (Bost et al., 2021; Ridge et al., 2017), together with seasonal patterns, are closely related to underlying population dynamics. We observed consistent patterns in median vertical reef growth and individual growth rates (Figure 3), aligning with known temperature‐controlled (Kamermans & Saurel, 2022) trends of seasonal growth periods (Diederich, 2006). Furthermore, detecting spat in subsequent autumn and spring samplings implies favourable conditions in the preceding year for growth and reproduction. The observed growth variations are consistent with established rates in the Wadden Sea, ranging between 26 and 46 mm yr^−1^ (0.07–0.13 mm d^−1^) (Schmidt et al., 2008), and are known to be primarily influenced by fluctuations in food availability and temperature (Diederich, 2006). Although food concentrations were not measured, population declines (high mortality) during the harsh winter of 2020–2021 may have led to reduced intraspecific competition for food resources, potentially improving growth conditions despite temperature constraints (Diederich, 2006; Rheault & Rice, 1996). Considering that biogenically induced vertical reef growth potentially did not exceed the maximum individual oyster growth (as overgrowth of other growing oysters did not contribute additionally), the highest observed median reef growth may also be related to individual growth rates. At KB, the period between March 2020 and March 2022 showed growth from 36 ± 12 mm to 104 ± 20 mm (Figure A3), an increase of 68 mm for the 2018 cohort, which is comparable to the observed maximum median reef growth of around 65 mm for the same period.

Beyond individual growth and abundance, spatial biomass dynamics and shell‐material supply to the reef structure play a pivotal role in spatial reef growth. However, our sampling design did not allow for the capture of cumulative shell accumulation as described by Walles, Mann, et al. (2015).

**FIGURE A4:**
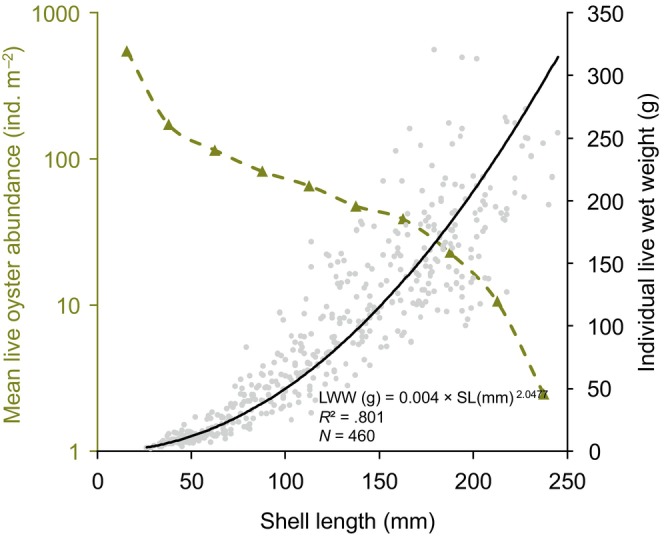
Relationship between *M. gigas* shell length (SL) (mm), mean live oyster abundance m^−2^ and individual live wet weight (LWW) (g) at the Kaiserbalje oyster reef. The dashed green line and triangles represent the trimmed mean oyster abundance m^−2^ for size classes from 2006 to 2022, with the highest and lowest 10% of mean values excluded. The scale for mean live oyster abundance is displayed logarithmically. The relationship between individual live wet weight and shell length (SL) is depicted by the solid black logistic regression line. Individual measurements of live wet weight are represented as grey dots.

The combination of individual growth rates and the starting population (at different lengths; Figure 3c–e) provides evidence of the increasing contribution of larger oysters to spatial growth dynamics, as proposed by a previous accretion study (Walles, Mann, et al., 2015). Additionally, a delayed effect of individual recruitment events is evident in the biomass progression of individual oysters and cohorts (Figures 7 and A4). Precise quantification of the exact relationships between individual biomass gain, decreasing cohort sizes and resulting reef expansion should be the focus of further investigations.

Similar to *M. gigas* populations in the Wadden Sea, *C. virginica* populations on the US East Coast display annual variations in recruitment, mortality and growth rates (Mann et al., 2009). Discrepancies between variations in annual spatial growth rates and patterns observed in environmental factors like temperature and salinity (Ridge et al., 2017) may be attributed to the delayed effects recruitment experienced several years prior to the study intervals. Moreover, annual volumetric growth differences, varying by a factor of two between years (Ridge et al., 2017), might be linked to the progression of cohort‐related biomass (Walles, Mann, et al., 2015), besides, for example, sediment dynamics in accretion studies. The categorization of oyster reefs on the basis of an age‐dependent maturity status, as implemented for *C. virginica* reefs (Bost et al., 2021), may not be directly applicable to oyster reefs in the Wadden Sea, especially if reefs show periods of accelerated growth interspersed with less active years.

The cumulative effects of oyster recruitment, growth and survival not only impact the amplitude of overall vertical reef growth but are also fundamental to the elevation‐related expression of an optimal growth zone and the positioning of the highest structural reef growth. Fodrie et al. (2017) made a substantial contribution to our understanding of reef growth dynamics by providing evidence that the optimal growth zone adapts over short periods of time (down to annually) to fluctuations in water level and corresponding exposure time. Fluctuations in mean water level and exposure time were recorded in our study area, with exceptionally high water levels in winter 2021–2022, likely caused by frequent storm surges with winds from west to northwest directions (German Meteorological Service (DWD), 2024). Comparing seasonal elevation and exposure time‐related vertical reef growth patterns at KB (Figure A2a,c), adjusting for exposure time of individual scan intervals revealed an overlap of positions of higher vertical reef growth, supporting findings by Fodrie et al. (2017). However, these short‐term shifts in water level and exposure time did not proportionally cause shifts in the position of maximum vertical reef growth, also seen at some *C. virginica* reefs (Fodrie et al., 2017). In our opinion, it is crucial to consider several population‐level implications in the assessment of vertical reef growth patterns in relation to water level fluctuations. Changes in water level and exposure time will initially cause a population‐level feedback by increasing or decreasing individual growth and shifting spatial oyster abundance due to changes in survival rates, which will subsequently lead to the expression of detectable elevation‐related vertical reef growth patterns and an optimal growth zone. As described by Walles et al. (2016) for *M. gigas*, after spawning events, juvenile oyster recruits showed an optimum around an exposure time of 36% MTR, indicating that water level and exposure gradients at the time of recruitment determine the initial elevation‐related oyster abundance. Subsequent shifts in water level affect individual oyster growth and survival pattern in relation to exposure time; however, these changes remain constrained by the initial recruitment pattern. Ridge et al. (2015) mentioned that for *C. virginica* reefs, adult oyster abundance can be higher above the optimal growth zone, highlighting the influence of both oyster abundance and individual growth on the optimal growth zone. A higher abundance of oysters above the optimal growth zone may be associated with a reduction in individual growth at higher elevations (Walles et al., 2016) (probably also accounts for *C. virginica*). Additionally, fluctuations in water level will affect different developmental stages of individual oyster cohorts in different ways. Adult oysters will benefit from a slight decrease in water level, as their survival increases at higher elevations due to their ability to cope better with aerial exposure compared to prolonged submersion. Juvenile oysters will benefit from a slight increase in water level as their survival is higher at lower elevations (Walles et al., 2016). Therefore, even when the water level remains constant but recruitment patterns fluctuate highly, one individual oyster cohort may shift a detectable structural growth pattern during their maturity. Initially, there might be higher structural reef growth at lower elevations in the first year or growth season after recruitment. In the following years, the structural reef growth may shift to slightly higher elevations due to shifts in survival rates of oysters with age. Therefore, it probably takes time before a detectable optimal growth zone develops, especially when oyster recruitment shows high fluctuations between years. This might be the case at the KB study site. Future research is needed to evaluate the specific population‐level feedback to fluctuating water levels and the resulting structural reef growth to enhance our understanding of these complex dynamics.

### 4.3 Internal reef morphodynamics

Besides the biogenic component, the spatial analysis of oyster reef growth is influenced by a variety of morpho‐ and hydrodynamic effects. At KB, shifts in the position of tidal creeks at parts of the reef margin result in destabilization of the reef framework and partial detachment of oysters. Reef internal drainage led to sediment erosion within the reef framework, causing small‐scale destabilization and subsidence of the reef surface. At NL, wave‐enforced rearrangement of loose oyster clusters was observed. In addition to reef growth of the oyster framework, reef internal sedimentation plays an important role in sustainable reef accretion. However, seasonal accumulation of biodeposits, that, faeces and pseudofaeces (Bungenstock et al., 2021), probably overestimates reef accretion rates. Irrespective of the effect that the biodeposits are stabilized by microbial mats during summer most of the material will be reworked during winter season and therefore contribute only to a minor amount to the sustainable vertical reef accretion. Fluctuations in large‐scale sedimentation and erosion patterns reveal previously covered reef areas, thus implying lateral and volumetric growth. However, uncovered none‐growing or dead reef areas are inactive unless recolonized by subsequent spatfalls. In this context, large‐scale sedimentation reduces visible reef area by burying parts of the reef margin and lower‐lying areas, while the remaining area potentially appears to accrete due to non‐sustainable reef‐internal sediment accumulation. These internal sediment accumulations within the reef can reach close to 150 mm, equivalent to the roughness height of the oyster reef surface (Hitzegrad et al., 2022) before the reef is completely covered and becomes unrecognizable as a reef structure. It is crucial to account for these factors when evaluating remote sensing 3D monitoring data to accurately distinguish between oyster reef growth and the accretion of the biosedimentary reef structures.

### 4.4 Methodological and systemic limitations

The restriction in the sampling of population data (12 stations with each 25 × 25 cm) does not fully allow to capture growth variations within the reef and the ROI in detail. The variable growth geometry of oysters, that is, long and narrow, where abundances are high, versus broad in marginal clusters (Hitzegrad et al., 2022), introduces additional variability into the length‐frequency distribution and biomass analysis. The lack of on‐site data loggers during our study period to record temperature and aerial exposure time was an additional limitation.

In terms of terrestrial laser scanning, the restricted size of the ROI represents the entire reef structure only to a limited extent. The delineation of sharply decreasing vertical reef growth towards the highest elevation within the ROI suggests that the upper spectrum of reef structures is adequately covered, with the highest elevation bin at KB for March 2020 at −0.19 m NHN comparable to drone‐based photogrammetry estimations of the reef top position. According to Hoffmann et al. (2023), the reef top position at KB was −0.155 ± 0.036 m NHN for March 2020 and −0.182 ± 0.027 m NHN for September 2020. The lower growth boundary may not be fully captured due to the limited extent of the ROI at lower elevations (Figure 5).

Previous studies using terrestrial laser scanning for evaluating vertical oyster reef growth did not encompass sub‐annual survey intervals due to uncertainties in the georeferencing process by RTK‐GPS (Bost et al., 2021; Ridge et al., 2015). The proposed method to align seasonal ScanScenes to a local coordinate system, based on reference spheres placed on iron rods, showed vertical uncertainties of <1 cm; for seasonal comparisons with median vertical M3C2 distances of 5.61–19.33 mm, most values lie within the vertical uncertainty. A cut‐off of the vertical M3C2 distance frequency distribution at a certain level above and below the uncertainty would increase the evidence for significant change interpretation; however, when the process that wanted to be observed (biogenic reef growth) lies with its median and IQR within the excluded part, the residual contains mainly extreme values. Predominantly those, which were not influenced by biogenic reef growth but account for morpho‐ and hydrodynamic effects.

Therefore, estimates of annual growth rates were made based on the vertical growth from the first to last observation over the period of 2 years for KB and 1.5 years for NL, which are considered to be most feasible for general interpretation and on which a pronounced elevation‐related vertical growth gradient with an optimal growth zone develops.

Although the seasonal ScanScenes were co‐registered in a single process, each seasonal comparison remained independent, utilized different Corepoints and varied locally in point densities. The low residuals between the comparisons of the median vertical M3C2 distances from the first to the last survey and the sum of seasonal medians, which were 3.37 mm for KB and 2.13 mm for NL, demonstrate the consistency of the proposed method, and potentially that seasonal vertical median values are feasible for interpretation.

The reference spheres used to establish a local coordinate system are crucial in the FARO Scene environment. However, due to time constraints imposed by the tides, only 12–13 fixed positions for reference spheres were feasible over an area of approximately 80 × 80 m. This limited number is not considered sufficient for excluding some positions from the registration and co‐registration process and using them exclusively as independent checkpoints for the estimation of an alignment error (Anders et al., 2020). In our study, the misplacement and the interpolation of sphere centroids introduced an additional degree of uncertainty. Generally, the use of reference sphere positions is not considered as accurate as the use of poles or rods as such, and the use of their 3D point cloud displacements for the calculation of alignment errors (Muralikrishnan et al., 2017). When the number of fixed reference positions is low as in our study, excluding reference positions for error calculation would potentially lead to low quality of spatial alignment. Bootstrapping the co‐registration process by excluding a low number of fixed reference spheres in each iteration from the co‐registration process and using them to calculate an alignment error would allow for more discrete estimations of errors. However, it is limited by software constraints and would drastically increase computing time and data storage.

On ultra‐rough surfaces such as oyster reefs, uneven shadow effects occur in the 3D point clouds when individual scan positions were not exactly reproduced during each survey. This limits the reliable use of the M3C2 method for providing roughness‐based error estimations (Lague et al., 2013).

### 4.5 Perspectives and controls in future reef growth

Climate change will have a multifaceted impact on *M. gigas* populations in the Wadden Sea and beyond. General warming trends are projected to facilitate the reproductive success of *M. gigas* in European coastal waters (King et al., 2021; Rinde et al., 2017; Teixeira Alves et al., 2021). Projections for the trilateral Wadden Sea region predict a rise in air temperature ranging from +0.9°C to +4.8°C, with a potential average shift of +2.6°C by 2100 and a projected increase in regional precipitation and in days with temperatures reaching or exceeding 30°C (hot days) and 25°C (summer days) (Norddeutsches Klimabüro, 2023). A rise in air temperature will highly impact the coastal water masses in the Wadden Sea region, with a water temperature increase expected to rise in the same order (1–5°C) (Kloepper et al., 2017). In general, extended summer periods and milder winters will increase spawning, leading to generally higher oyster abundances on the reef structures. Although higher abundances can stabilize and enhance vertical reef growth rates and population resilience, intraspecific competition, potential heat stress and emerging diseases (Reise, Buschbaum, Büttger, Rick, et al., 2017; Walles, Mann, et al., 2015) may counteract these benefits. For the 3D habitat complexity and function, the existing variability in oyster recruitment and shell length distribution up to 250 mm is essential (Hesterberg et al., 2020). The frequent occurrence of recruitment events and potentially shorter lifespans among oysters (Walles, Mann, et al., 2015) may lead to a reduction in this structural complexity by depleting larger structural components. Such a reduction in the shell length distribution would also reduce the topographical roughness (Hitzegrad, Brohmann, et al., 2024; Hitzegrad, Köster, et al., 2024) and thus the interactions with the ambient hydro‐ and morphodynamics. Warming water masses will also influence interspecific interactions; for instance, a decrease in *M. edulis* populations (Philippart & Epping, 2009) within reef structures which have a lower temperature optimum than *M. gigas* (Kamermans & Saurel, 2022) and higher survival rates of predators during milder winters (Philippart et al., 2003) could modify predator–prey dynamics and might lead to a rise in predation on *M. gigas* larvae. Furthermore, the occurrence of toxic algal blooms (Karlson et al., 2021) and oxygen minimum conditions are additional factors that will have an impact on the Wadden Sea ecosystem in general and specifically on *M. gigas* under future warming trends. Consequently, the condition of *M. gigas* populations and reef resilience is not solely dependent on *M. gigas* species‐specific ecological tolerances dynamics. Understanding its development also necessitates a comprehensive evaluation within the context of ecological network analyses.

In terms of sea level rise, both investigated reefs have already demonstrated resilience to the observed rise in mean sea level from 1993 to 2011, which was 2.2 ± 2.5 mm yr^−1^ for Norderney (15 km east of NL) and 3.7 ± 2.3 mm yr^−1^ for Cuxhaven (40 km east of KB) (Wahl et al., 2013). Our results of median and peak growth rates suggest a robust potential for resilience against current and projected rates of sea level rise. Most anticipated projections of the local sea level rise for the study area for 2100 relative to 2000 range from 39 to 43 cm sea level rise (66% uncertainty interval: 35–90 cm) for a scenario with end‐of‐century warming below 2°C to 70–75 cm sea level rise (66% uncertainty interval: 62–148 cm) for a scenario with end‐of‐century warming exceeding 4°C (for comprehensive local projections, refer to Climate Analytics, 2018. Local sea level rise. Available at: https://localslr.climateanalytics.org) (Bamber et al., 2019; Kopp et al., 2014; Rasmussen et al., 2018).

However, the coastal geometry and bathymetry of back‐barrier areas and ebb‐tidal deltas in the Wadden Sea will lead to complex feedback between sea level rise, tidal amplitudes and morphodynamic patterns (Hagen et al., 2022; Jordan et al., 2021). This feedback will affect local growth ceilings in relation to exposure time and could significantly modify the direct impact of sea level rise. As observed growth rates at the reef crest at both reefs approach values of the local sea level rise, an impending temporal equilibrium can be assumed. As evidenced by increasing growth rates from the reef crest to the optimal growth zone (Figure 5), a faster sea level rise would most likely lead to enhanced growth, stabilizing at a new equilibrium (Ridge et al., 2017) at the reef crest. Vertical growth exceeding sea level rise on the reef flanks fosters the development of plateau‐like elevation gradients (Ridge et al., 2015). Steeper reef flanks enhance hydrodynamic forced dispersal of shell material (Lukasik & Simo, 2008), probably facilitating lateral propagation of the reefs, particularly as hard substrate is limited on the tidal flats.

Additionally, projections of vertical reef growth related to sea level rise need to take related shifts in local morphodynamic patterns and sedimentation rates into account (Ridge et al., 2015; Rodriguez, 2017). Observed sedimentation rates were around 5.1–7.5 mm yr^−1^ in the tidal basin of NL and 7.6–10 mm yr^−1^ for the KB area between 1998 and 2016 (Benninghoff & Winter, 2019). Although both reefs demonstrated resilience to the observed rates, an increase in these local sedimentation rates could reduce reef areas from lower marginal areas upward. The local migration and relocation of tidal channel systems (Bungenstock et al., 2021) can also easily rework the reef structures, as observed by the lateral migration of the eastern tidal channel at KB.

## 5 CONCLUSION

This study highlights the link between seasonal population dynamics and the resulting vertical reef growth of two *Magallana gigas* reefs in the central Wadden Sea. The intertidal reefs were analysed with respect to their short‐term (2 years) seasonal vertical growth patterns by terrestrial laser scanning and the population dynamics of the reef‐building oysters based on an established monitoring design.

To minimize spatial alignment uncertainties, we established a local coordinate system based on reference spheres placed on iron rods deeply penetrated into the sediment. This allowed us to limit the overall vertical root mean square of reference sphere positions to 8 mm for KB and 5 mm for NL.

Median vertical reef growth of 19.8 mm yr^−1^ for KB and 17.5 mm yr^−1^ for NL were quantified, with seasonal rates of 0.03–0.05 mm d^−1^ in winter and 0.06–0.09 mm d^−1^ in summer. These seasonal variations were closely associated with the temperature‐related growth activity of *M. gigas*. The mean oyster abundance on KB was 627 ± 43 ind. m^−2^ and 338 ± 87 ind. m^−2^ on NL, corresponding to biomass values of 31.1 ± 3.5 kg LWW m^−2^ in KB and 19.0 ± 2.7 kg LWW m^−2^ in NL. In addition to individual oyster growth, the seasonal data on population dynamics, mainly shell length and abundance, reveal that the presence of larger individuals has positive feedback on vertical reef growth. Therefore, strong recruitment triggered by long and warm summer periods has a delayed effect on enhanced vertical reef growth.

The observed median vertical reef growth rates highlight the potential resilience of these reefs in the face of expected accelerated sea level rise, exceeding current local rates in sea level rise of 2.5 and 3.7 mm yr^−1^ (Wahl et al., 2013). The minimal growth observed in the central reef crest area suggests that their vertical positioning relative to the current sea level has reached a state of equilibrium and thus the limitation by its ecological tolerance to tidal emergence. Based on local tide gauge data, the reef crest at KB is at 42.1% and at NL at 42.2% measured exposure time. Considering the local mean tidal range, the reef crest at KB is at 47.8% and at NL at 48.4% exposure time. Higher vertical reef growth in the lower lying parts of the reef reflects the optimal growth zone. The data of vertical reef growth from our study are similar to those from previous surveys along the US East Coast (*Crassostrea virginica*, maximum median reef growth rates from 28.9 to 66.7 mm yr^−1^) (Ridge et al., 2017) and the southern North Sea (Oosterschelde, *Magallana gigas*, long‐time accretion rates from 7 to 16.9 mm yr^−1^) (Walles, Mann, et al., 2015).

Seasonal growth patterns on both, individual oysters and reef structures provide essential insights for assessing future populations and reef resilience under shifting climatic conditions. However, detecting sub‐annual growth patterns remains challenging due to methodological limitations like accuracy, limited tidal time windows and the inherent flexibility of the oyster reef framework.

The prospective growth and stability of existing reefs will not solely depend on the reproductive output of the reef‐building oysters but also on local hydro‐ and morphodynamic feedback in relation to sea level rise. Estimating reef‐specific resilience, therefore, needs to incorporate species‐specific ecological tolerances with the stability and 3D propagation of oyster reefs as biosedimentary facies bodies.

## AUTHOR CONTRIBUTIONS

**Kai Pfennings:** Conceptualization (equal); data curation (lead); formal analysis (lead); investigation (equal); methodology (lead); visualization (lead); writing – original draft (lead); writing – review and editing (lead). **Tom K. Hoffmann:** Investigation (equal); writing – review and editing (equal). **Jan Hitzegrad:** Investigation (supporting); project administration (supporting); writing – review and editing (equal). **Maike Paul:** Conceptualization (equal); funding acquisition (equal); investigation (supporting); project administration (equal); supervision (equal); writing – review and editing (equal). **Nils Goseberg:** Conceptualization (equal); funding acquisition (equal); project administration (lead); supervision (equal); writing – review and editing (equal). **Achim Wehrmann:** Conceptualization (equal); funding acquisition (equal); investigation (equal); project administration (equal); supervision (lead); writing – original draft (supporting); writing – review and editing (equal).

## FUNDING INFORMATION

The project BIVA‐WATT on which this work is based was funded by the Federal Ministry of Education and Research of Germany (BMBF) under the funding code 03KIS129.

## CONFLICT OF INTEREST STATEMENT

The authors declare that the research was conducted in the absence of any commercial or financial relationships that could be construed as a potential conflict of interest.

## Supporting information


Appendix S1

Appendix S2

Appendix S3

Appendix S4

Appendix S5

Appendix S6

Appendix S7

## Data Availability

Terrestrial laser scanning data used in this study are accessible from the DRYAD repository at https://doi.org/10.5061/dryad.j3tx95xq7. A summary of elevation‐related vertical reef growth is included in the Supporting Information – Appendix S1. Data of terrestrial laser scanning uncertainty estimations are included in the Supporting Information – Appendix S2. Data on population dynamics are included in the Supporting Information – Appendices [Link], [Link], [Link]. Data on water temperature are included in the Supporting Information – Appendix S6. Data from OLS regressions are included in the Supporting Information – Appendix S7.

## APPENDIX A

**TABLE A1:** Comprehensive summary of terrestrial laser scanning survey data for the oyster reefs Kaiserbalje and Nordland over multiple survey intervals.

Oyster reef	Kaiserbalje (KB)				Nordland (NL)				
Survey interval									
Week of first scan dd/mm/yyyy	02/03/2020	07/09/2020	15/03/2021	11/10/2021	02/03/2020	21/09/2020	19/04/2021	13/09/2021	21/09/2020
Week of second scan dd/mm/yyyy	07/09/2020	15/03/2021	11/10/2021	21/03/2022	21/03/2022	19/04/2021	13/09/2021	25/04/2022	25/04/2022
Days between measurements	189	189	210	161	749	210	147	224	581
Water level									
Mean (m NHN)	0.01	0.04	0.05	0.2	0.07	0.07	0.02	0.18	0.05
Std. deviation (m NHN)	1.22	1.16	1.20	1.21	1.20	0.99	0.88	0.90	0.93
M3C2									
Matching ROI corepoints	346,725	308,714	296,793	495,553	410,184	740,232	502,646	442,616	651,493
Corepoints >4 points in each ScanScene	258,964	265,732	253,681	449,831	320,197	521,805	309,294	305,083	401,041
Median (mm)	10.78	8.33	19.33	5.61	40.68	9.48	10.62	9.95	27.92
Mean (mm)	12.50	9.12	19.71	5.60	41.42	9.97	11.53	9.77	29.03
Std. deviation (mm)	18.80	13.85	15.55	14.97	23.86	17.47	17.27	16.77	25.74
Variance	0.35	0.19	0.24	0.22	0.57	0.31	0.30	0.28	0.66
25th percentile (mm)	0.81	0.72	9.81	−2.34	25.87	0.66	1.16	1.05	13.83
75th percentile (mm)	21.83	16.55	29.22	13.83	56.03	18.45	21.10	18.68	43.50
5th percentile (mm)	−14.09	−11.10	−4.55	−17.87	4.46	−15.84	−14.11	−17.01	−8.95
95th percentile (mm)	46.31	31.50	45.33	28.83	81.92	37.45	39.51	36.08	71.30
Skewness (−)	0.7595	0.8842	0.1275	−0.2079	0.1443	0.7934	0.8527	−0.1882	0.1565
Kurtosis (−)	2.6189	6.2006	1.3720	4.9506	0.9093	8.6311	8.0095	3.5933	3.0595
Daily vertical growth rate (mm d ^−1^	0.06	0.04	0.09	0.03	0.05	0.05	0.07	0.04	0.05
Reference uncertainty									
3D RMS of reference spheres (mm)	14.2	12.7	16.0	18.1	20.4	26.4	9.4	8.5	27.4
Lateral RMS of reference spheres (mm)	12.9	7.6	15.0	16.8	18.5	26.1	7.6	7.1	26.9
Vertical RMS of reference spheres (mm)	6.0	10.2	5.3	6.8	8.4	3.8	5.4	5.2	5.3
